# Fear and Challenging Behaviour: A Phenomenological‐Hermeneutic Study of Public Mass Shooting Attacks During the COVID‐19 Pandemic in Thailand

**DOI:** 10.1002/nop2.70398

**Published:** 2026-01-05

**Authors:** Ek‐Uma Imkome

**Affiliations:** ^1^ 99 Faculty of Nursing Thammasat University Klong‐Luang Patumtanee Thailand

**Keywords:** coping strategies, COVID‐19, fear, mass shootings, nursing care, phenomenology, psychological adaptation, qualitative research, social support, trauma‐informed practice

## Abstract

**Aim:**

To explore the lived experiences of Thai participants of public mass shooting during the COVID‐19 pandemic.

**Design:**

A phenomenographic research approach was used.

**Methods:**

Fifteen participants were recruited using purposive and snowball sampling. Data were collected through dialogical interviews and analysed using thematic analysis within a Heideggerian interpretive framework. The study adhered to the Consolidated Criteria for Reporting Qualitative Research (COREQ).

**Results:**

Through a hermeneutic lens, five interpretive themes were identified: dwelling in the shadow of threat, bearing witness to collective rupture, embodied echoes of trauma, grounding the self through everyday rituals and yearning for attuned care. These themes illuminate survivors' meaning‐making amid dual crises and reflect the complex interplay of somatic, psychological and social adaptation.

**Conclusion:**

The narratives of survivors underscore the urgent need for trauma‐informed, relationally grounded nursing care in the aftermath of mass shooting incidents. The five emergent themes—ranging from embodied fear to the yearning for attuned care—highlight the complex interplay of psychological, social and existential dimensions of trauma. These findings emphasise the importance of holistic, context‐sensitive interventions that not only validate survivors' emotional experiences but also foster adaptive coping and social reintegration. By recognising the embodied nature of fear and addressing survivors' multifaceted needs, healthcare professionals can play a pivotal role in facilitating recovery and promoting long‐term well‐being.

**Patient or Public Contribution:**

Fifteen individuals with firsthand experience of mass shootings during the COVID‐19 pandemic contributed personal narratives that informed the study's thematic analysis and nursing implications.

## 1 Introduction

Public mass shootings are a critical global public health concern, ranking among the leading causes of death due to injury in many regions (Centers for Disease Control and Prevention [CDC] 2019a, 2019b). These incidents are not only life‐threatening but also result in profound biological, psychological and social impacts on survivors and communities. The COVID‐19 pandemic exacerbated these challenges, leading to increased stress, social isolation and vulnerability among affected populations (Hossain et al. 2020; Hertz‐Palmor et al. 2021). During this period, the global surge in mass shootings raised urgent questions about the compounded effects of such crises on public health and safety (Kim and Phillips 2021).

Thailand, which has one of the highest rates of gun ownership in Southeast Asia—with over 10 million privately owned firearms—faces unique challenges related to mass shootings (Serhan 2022). The firearm‐related death rate in Thailand is approximately 7.2 per 100,000 individuals, significantly surpassing regional averages (Institute for Criminal Policy Research 2019). Recent incidents, such as the tragic shooting at a childcare centre in 2021, which claimed 37 lives, predominantly children (Maishman and Jeremy 2022), and the Siam Paragon mall shooting in 2023, highlight the devastating consequences of gun violence (Olarn and Stapleton 2023). Such events have far‐reaching implications for public safety and mental health, underscoring the urgency of addressing this crisis (Breiner 2023).

Mass shootings not only cause immediate physical harm but also result in long‐term psychological distress. Survivors frequently experience post‐traumatic stress disorder (PTSD), depression and social withdrawal, while indirect exposure through media can amplify community‐wide fear and anxiety (Bharadwaj et al. 2021; Shultz et al. 2014). Research indicates that prolonged exposure to traumatic events during the pandemic worsened these mental health outcomes, particularly among vulnerable populations such as children and the elderly (Peña and Jena 2021; Hahm et al. 2021).

Although existing literature has extensively documented the psychological consequences of mass shootings—including post‐traumatic stress disorder, depression and anxiety (Bharadwaj et al. 2021; Shultz et al. 2014)—and the mental health impacts of the COVID‐19 pandemic (Hossain et al. 2020; Hertz‐Palmor et al. 2021), there remains a notable gap in understanding how individuals construct meaning from their lived experiences when these two crises converge. Most studies have predominantly focused on clinical outcomes within a Western context, with limited attention to the sociocultural dynamics that influence trauma recovery in Southeast Asia. In particular, the Thai context—characterised by high firearm ownership and recent mass shooting incidents (Serhan 2022; Olarn and Stapleton 2023)—has not been adequately explored through qualitative inquiry. Furthermore, the compounded effects of pandemic‐related stressors, such as social isolation and restricted access to mental health services, present unique challenges that have yet to be examined in depth (Campedelli et al. 2020; Kamberi et al. 2021). This study addresses this critical gap by employing a phenomenological‐hermeneutic approach to investigate the lived experiences of Thai survivors of public mass shootings during the COVID‐19 pandemic, thereby contributing context‐sensitive insights to inform trauma‐informed nursing interventions and policy development.

## 2 Research Question

What are people's experiences with public mass shooting attacks during the COVID‐19 pandemic?

## 3 Research Objectives

This study aims to explore the lived experiences of Thai participants of public mass shootings during the COVID‐19 pandemic in Thailand.

The specific objectives

- To describe participants' perceptions of persistent fear and threat following the incident.
- To examine the role of shared emotional experiences and social connection in coping.
- To identify the physical, psychological, social and economic impacts of trauma.
- To explore personal coping strategies and everyday rituals that support recovery.
- To understand participants' expectations and experiences of healthcare and nursing support.

## 4 Methods

### 4.1 Ethics and Consent

This study was approved by the Human Research Ethics Committee of Thammasat University (Science) (HREC‐TUSc), Thailand (Approval No.: COA No. 119/2563) on October 20, 2022. Before data collection, all participants were thoroughly informed about the study's scope, including its purpose, potential risks and benefits. Written and verbal informed consent was obtained to ensure participants' understanding and voluntary agreement to participate in the study.

To safeguard participant well‐being, measures were taken to minimise psychological risks during and after the interviews. For participants experiencing distress, referrals to appropriate mental health services were readily available. All data collection processes were conducted in a private and secure virtual environment to ensure confidentiality and anonymity. Participants retained the right to withdraw from the study at any time without penalty, including requesting the removal of their data after the interviews. Measures to maintain anonymity and confidentiality included the deletion of video interviews from Microsoft Teams within one month after collection and secure storage of transcripts on encrypted hard drives. Data will be permanently deleted using Secure Deletion Shredder for Windows 10 three years post‐study completion, adhering to privacy standards (Tong et al. 2007).

### 4.2 Design

This study utilised a phenomenological‐hermeneutic design grounded in Heideggerian philosophy, which foregrounds the interpretive nature of human experience and the inseparability of individuals and their sociocultural and historical contexts. As a methodology familiar within the social sciences, this approach was particularly appropriate for exploring how individuals construct meaning through lived experience.

The approach enabled an in‐depth examination of participants' narratives concerning public mass shooting incidents during the COVID‐19 pandemic. Employing a narrative‐oriented framework aligned with a recovery‐oriented paradigm, this method facilitated interpretations of personal accounts within broader existential and relational dimensions (Kvale and Brinkmann 2014; Kirkpatrick 2008).

Guided by Heidegger's concept of being‐in‐the‐world, the study prioritized idiographic depth, concentrating on the contextualized nature of lived fear, relational vulnerability and bodily disruption. This ontological perspective informed both data collection and analysis, allowing existential meanings to emerge inductively from participants' narrative expressions.

### 4.3 Setting and Participants

The study was conducted in XXX Province, a region directly impacted by one of the country's high‐profile mass shooting events.

Participants were recruited using purposive and snowball sampling techniques to ensure variation in demographic characteristics and experiential backgrounds. Inclusion criteria required participants to be at least 18 years old, have direct exposure to the mass shooting (either as survivors or witnesses), and the capacity to provide informed consent. Individuals experiencing acute psychiatric instability or cognitive impairments were excluded to protect emotional and ethical integrity during data collection (Smith, Silva, et al. 2020; Smith, Sharpe, et al. 2020).

### 4.4 Study Measures

#### 4.4.1 Semi‐Structured Interview Guideline

The semi‐structured interview guideline (Imkome 2024a, 2024b, 2024c) was meticulously developed to elicit deep, reflective narratives, consistent with phenomenological‐hermeneutic inquiry. It underwent expert review by three qualitative scholars to ensure content validity and philosophical alignment. The guide featured nine open‐ended, interpretive prompts designed to uncover participants' lived experiences, perceptions of meaning and embodied responses to public mass shootings during the COVID‐19 pandemic.

Rather than merely soliciting factual information, the questions were crafted to invite existential reflection, emotion‐laden memory and meaning‐making processes. For instance:

Can you describe what it felt like to be present during the mass shooting event?

In what ways has that experience affected your sense of safety, self, or relationships?

What does coping look like for you after what happened?

How have the responses from healthcare providers shaped your healing or recovery?

While nine anchor questions provided structure, the interview process remained fluid and responsive, allowing the interviewer to follow the participant's narrative direction. This flexibility honoured the idiographic and interpretive nature of phenomenological research by enabling participants to lead with their meaning systems.

### 4.5 Data Collection

Data were collected between December 2020 and March 2021, coinciding with XXX's second wave of COVID‐19, a period marked by heightened public health restrictions and partial lockdowns. This conditions shaped participants' lived experiences and influenced their access to support systems. Participants were recruited using purposive and snowball sampling to ensure diversity in demographic and experiential backgrounds. Prior to interviews, each participant completed a structured demographic questionnaire capturing variables such as age, gender, marital status, educational level, occupation, income, health insurance coverage, chronic health conditions and living arrangements. This contextual information enriched the interpretive depth of the narratives and supported a nuanced understanding of individual experiences.

Data were collected through dialogical interviews with 15 individuals. Sampling continued until thematic saturation was achieved, defined as the point at which no new patterns or theme were identified in subsequent interviews (Braun and Clarke 2006). Interviews were conducted by the principal investigator—a female researcher with a Ph.D. and over 25 years of experience in psychiatric and mental health nursing—alongside trained research team members. Participant contact information was obtained through collaboration with nurses across various healthcare settings in the study region.

Potential participants were contacted by phone and invited to participate. Prior to the interviews, the research team disclosed their assumptions and personal interest in the topic to promote transparency. Written and verbal informed consent were obtained from all participants. Interviews were scheduled during participants' days off to minimise disruption and were conducted individually in the local language using a semi‐structured format.

To ensure safety during the pandemic, all interviews were conducted virtually via Microsoft Teams. Only the interviewer and participant were present during each session. Interviews lasted between 20 and 60 min and were securely recorded using the platform's built‐in software. The interviewer maintained a professional and neutral stance throughout the process.

Sampling continued until thematic saturation was achieved (Braun and Clarke 2006). Interview transcripts were anonymized and returned to participants for member checking, allowing for validation and correction prior to final analysis.

### 4.6 Data Analysis

Thematic analysis, guided by Braun and Clarke's reflexive methodology, was adapted within a phenomenological‐hermeneutic framework to preserve idiographic richness while enabling pattern recognition. The analysis remained consistent with Heideggerian inquiry by privileging the emergence of existential meaning through deep immersion in participants' narratives. The following steps were undertaken to ensure analytical rigour and philosophical alignment:

1. Familiarisation: The author read each transcript multiple times to gain a deep understanding of emotional tone, recurring language and embodied expressions of trauma.
2. Initial coding: Initially coding was performed using Microsoft Excel to organise emerging concepts such as ‘emotional response’, ‘support system’ and ‘economic strain’.
3. Theme development: Codes were iteratively reviewed and clustered into broader interpretive themes reflective of the participants' meaning‐making, such as dwelling in the shadow of threat and yearning for attuned care.
4. Theme refinement: Theme were refined to ensure internal coherence and analytical resonance.
5. Naming and Interpretation: Theme names were guided by phenomenological orientation, emphasising existential meaning over descriptive categorization.
6. Illustration: Representative quotations were selected to illustrate essential patterns while preserving the idiographic depth of personal narratives.

## 5 Results

### 5.1 Demographic Data of Participants

The study included 15 participants, most of whom were male (66.67%) and between the ages of 41–50 years (46.67%), followed by those aged 31–40 years (26.66%). One‐third of the participants (33.33%) were single, and 66.67% had attained a bachelor's degree or higher. The largest occupational group comprised government or state enterprise employees (40%), followed by individuals engaged in personal business or trading (26.67%). Regarding health coverage, 40% were enrolled in the Universal Health Coverage Scheme, while 20% were covered by the Social Security Scheme (Table 1).

**TABLE 1:** Demographic of participants experiencing public mass shooting attacks during the COVID‐19 pandemic (*N* = 15).

Demographic data	*N* (15)	%
1. Age		
1. < 30 years	3	20
2. 31–40 years	4	26.66
3. 41–50 years	7	46.67
4. 51–60 years	1	6.67
2. Gender		
1. Female	5	33.33
2. Male	10	66.67
3. Marital status		
1. Single	5	33.34
2. Couple	4	26.66
3. Widow	3	20
4. Divorced/separated	2	13.33
5. Separate	1	6.67
4. Level education		
1. Primary school	2	13.33
2. Secondary education	2	13.33
3. Vocational Certificate/Higher Vocational Certificate/Diploma	1	6.67
4. Bachelor's degree or higher	10	66.67
5. Career		
1. Government service/state enterprise employee	6	40
2. Trading or running a personal business	4	26.67
3. Unemployed	3	20
4. Employed	2	13.33
6. National Health Security		
1. Universal Health Insurance (Gold Card)	6	40
2. Social Security Scheme	3	20
3. Government or State Enterprise Officer	6	40
7. Chronic conditions or comorbidities		
1. No	7	46.66
2. Yes		
Depression	4	26.67
Diabetes and hypertension	4	26.67
8. Family member		
1. Father	9	60
2. Mother	10	66.67
3. Elder sibling	4	26.67
4. Sibling	2	13.33
5. Friend	2	13.33

In terms of health status, 46.66% reported no chronic conditions, whereas 26.67% disclosed diagnoses such as depression, diabetes, or hypertension. To better reflect the nature of these conditions, the term ‘chronic conditions or comorbidities’ was used in place of ‘congenital disease’. Additionally, participants identified key household or support figures—such as parents, siblings, or close friends—which provided insight into their social support networks and living arrangements.

### 5.2 Main Themes

**FIGURE 1:**
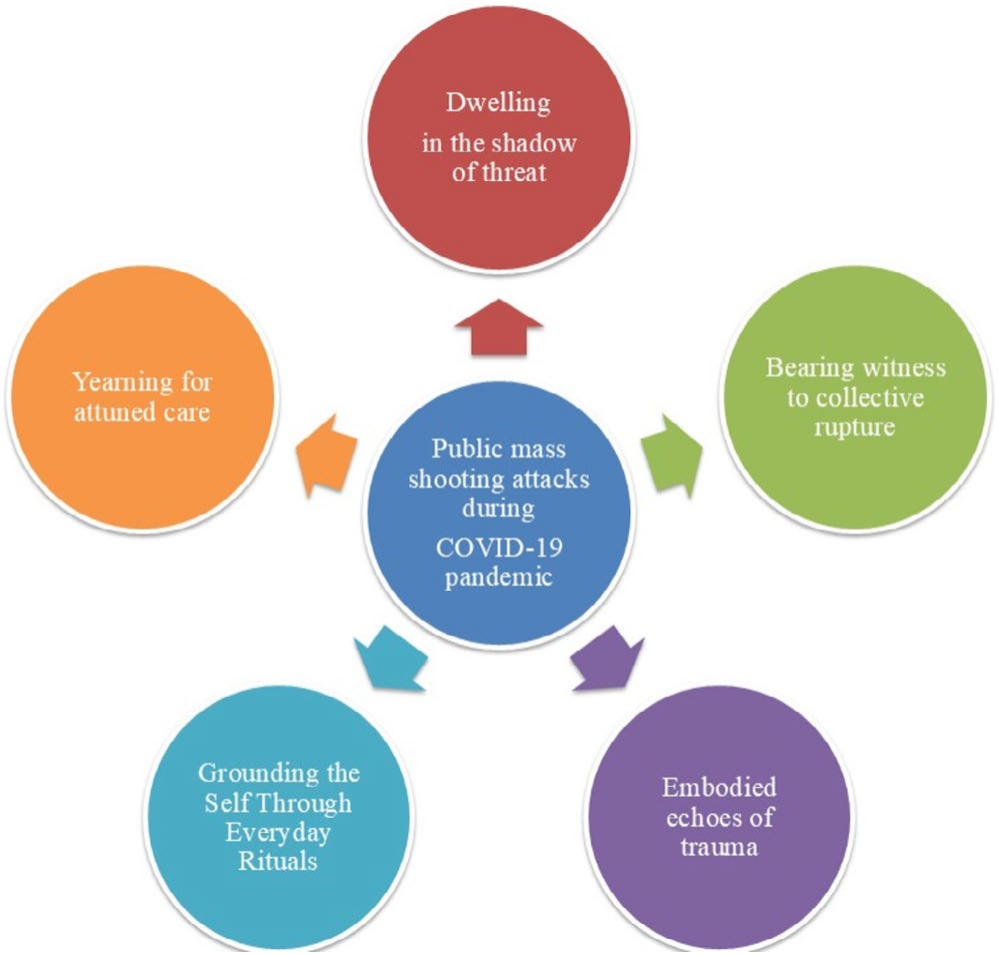
Theme of the study.

The results of in‐depth interviews with groups that experienced mass shootings during the COVID‐19 pandemic may be summarised in five main themes (Figure 1):

#### 5.2.1 Dwelling in the Shadow of Threat

Participants recounted a pervasive, inescapable sense of fear—an emotional atmosphere that lingered long after the incident, shaping perceptions of safety and embodiment.

##### 5.2.1.1 Emotional Response to Threat

The participants illustrate how individuals perceive imminent threats and danger, leading to feelings of terror, fright and anxiety. This highlights how fear can manifest physically and psychologically, affecting one's sense of safety and security. Some participants stated:

I was terrified. (Cl.MS 1)

##### 5.2.1.2 Impact on Daily Life

Fear can significantly disrupt normal routines and affect daily activities, as individuals struggle to cope with the aftermath of traumatic events. The testimonies reveal a conflict between the desire to return to normalcy and the overwhelming fear that prevents it. The following remarks were shared by participants:

I went to work as usual, but this happened. (Cl.MS 1)

##### 5.2.1.3 Survival Instincts

Actions to self‐protect emphasised the primal nature of fear, such as barricading themselves during the mass shooting attack. Participants expressed the following perspectives:

I thought I had to do something, so I went to take down both wheels and lock the room. (Cl.MS 6).

These facets of fear underscore its complexity, revealing how it shapes behaviour, influences decision‐making, and affects mental health following traumatic events. The sub‐theme also emphasises the importance of social support and coping strategies in mitigating fear responses.

#### 5.2.2 Bearing Witness to Collective Rupture

Bearing witness to collective rupture refers to sharing an unavoidable situation. It highlights the shared experience of individuals facing a traumatic event, such as a mass shooting attack, emphasising unity, collective fear, and the emotional support derived from being in a group during a crisis. The following are the sub‐themes:

##### 5.2.2.1 Shared Fear and Vulnerability

Individuals experience a collective sense of fear and vulnerability when confronted with danger. The shared environment amplifies their fear while also fostering a sense of camaraderie. Some participants noted that:

As soon as I heard the gunshot, I ran to the women's restroom… 20 people here, including children. They feared the children would cry and the culprit would walk up. (Cl.MS 1)

When the shooting started, I felt like the walls were closing in on us. We were all scared, but it was comforting to know others were just as terrified. (Cl.MS 5)

There were about 20 people with us… After a few hours of waiting, we began to feel it was time to relax and sit together. As we talked about our lives, we came to realize that we were all facing something unimaginable, which uniquely brought us closer together. (Cl.MS 7)

##### 5.2.2.2 Emotional Support Through Connection

The presence of others provides emotional support, which can be crucial during life‐threatening situations. Individuals highlight the importance of supporting and comforting one another amidst chaos. As mentioned by certain participants:

When I was depressed… when we encountered this incident, it made me see many truths: I don't want to die. (Cl.MS 4)

In that moment, I held the hand of the stranger next to me. We didn't know each other, but we both needed that support. It somehow made everything feel a little more bearable. (Cl.MS 6)

##### 5.2.2.3 Clinging to Connection in Uncertainty

Staying connected through communication tools like LINE becomes vital for updating each other about safety and needs (e.g., hunger or thirst). This indicates how technology can play a role in survival and emotional reassurance. Some participants shared the following insights:

We communicated by LINE application… if you were thirsty, turn on the tap water and drink it. (Cl.MS 2)

I kept texting my family updates, trying to reassure them. It felt good to share what was happening, and somehow it made me feel less alone. (Cl.MS 3)

##### 5.2.2.4 Collective Survival Instinct

The instinct to survive becomes a shared goal among those affected, creating a sense of teamwork and cooperation. Individuals work together to find safety and encourage each other. The following statements were made by participants:

… we had to escape from there and encourage each other. (Cl.MS 2)

Everyone in the restroom knew we had to stick together. We decided to stay put and wait for help instead of trying to escape alone. (Cl.MS 6)

##### 5.2.2.5 Understanding the Value of Life

The experience fosters a deeper appreciation for life and connections to others, as individuals recognise that they are part of a larger community facing the same threat. Participants reported the following observations:

… I realized that 21 people were living together in that room. Everyone had a family and a future as well. (Cl.MS 4)

Hiding there made me reflect on what really mattered—it wasn't just about surviving the shooting, but also about cherishing the ones we love. (Cl.MS 4)

##### 5.2.2.6 Uncertainty in Crisis

The confusion and disorder during the crisis highlight the uncertainty that everyone feels, yet this shared confusion can enhance the sense that they are not alone in their fear. Certain participants conveyed the following perspectives:

We couldn't see what was happening outside, and the uncertainty was terrifying. But since we were all in the same boat, we tried to stay calm together. (Cl.MS 5)

I did not know which way to run. (Cl.MS 7)

These quotes further illustrate how individuals navigate their shared experiences during a traumatic event, highlighting the themes of connection, emotional support, and the importance of community in coping with fear and uncertainty.

#### 5.2.3 Embodied Echoes of Trauma

‘Embodied echoes of trauma’ refers to the impact of a mass shooting incident that encompasses various dimensions, including physical, mental, social and economic aspects, significantly altering the daily lives of victims. Key sub‐themes are outlined below:

##### 5.2.3.1 Physical Impact

This refers to changes in physical well‐being, such as sleep disturbances and auditory hallucinations. Participants reported difficulty sleeping due to flashbacks and auditory hallucinations related to the shooting incident. For example:

When I sleep, I am frightened when I hear something. I cannot sleep. It is a flashback, and I always hear a gun sound (Cl.MS. 1).

##### 5.2.3.2 Mental Impact

This includes psychological trauma, such as persistent fear, anxiety and startle responses to reminders of the incident. Participants expressed:

After the incident occurred around February, the sound sounded like a bang; It startled me all the time. (Cl.MS. 6)

##### 5.2.3.3 Social Impact

This involves alterations in social behaviour, such as avoiding certain places or activities due to traumatic associations. Some participants stated:

I cannot go to Foodland alone. I must go with friends. I was dizzy. The smell of blood was overwhelming. I was an employee who had a house next to the terminal. (Cl.MS. 1)

##### 5.2.3.4 Economic Impact

This refers to disruptions in employment or the work environment, affecting the victims' economic stability. Participants illustrated:

I was an employee who had a house next to the terminal. (Cl.MS. 1).

This structured approach highlights the multifaceted nature of the impact experienced by victims of mass shootings, providing a comprehensive understanding of their challenges.

### 5.3 Grounding the Self Through Everyday Rituals

‘Grounding the Self Through Everyday Rituals’ refers to the various strategies employed to mitigate stress and trauma after experiencing a mass shooting situation. The sub‐themes presented are:

#### 5.3.1 Recreational Activities

Participants find solace in activities like gardening, arts and watching movies and TV series, which provided a therapeutic outlet to manage their stress and anxiety. The participant stated:

I do not sleep well. When I sleep, I am frightened. I woke up and went into the garden to do some embossing. Being close to brothers and sisters is better. (Cl.MS. 1)

Watch movies, reduce your media consumption. Inhale and exhale. Know your consciousness. (Cl.MS. 2)

Exercise, watch movies, listen to music, watch television series. (Cl.MS. 6)

#### 5.3.2 Social Connections

Family and friends played a crucial role in emotional recovery. As one participant mentioned:

Being close to brothers and sisters is better. (Cl.MS. 1)

Mindfulness and Relaxation Techniques: Techniques like breathing exercises and mindfulness help maintain mental equilibrium. One participant noted:

Inhale and exhale. Know your consciousness. (Cl.MS. 2)

This structured approach highlights the various coping strategies employed by individuals who have experienced a mass shooting, providing a comprehensive understanding of their methods for dealing with trauma.

#### 5.3.3 Yearning for Attuned Care

‘Yearning for attuned care’ refers to the diverse activities through which shooting victims receive support from the health team, encompassing physical, mental, social and economic assistance. Sub‐themes explored include.

##### 5.3.3.1 Therapeutic Activities

Group therapy, art therapy sessions and counselling were particularly beneficial. This activities providing a space for victims to share their feelings and experiences with others who have gone through similar trauma. Participants noted:

The hospital team took us to do activities, such as drawing a picture from your imagination. Asked why you drew it, you were invited to draw it again. How did you feel when the incident happened at the terminal? And after the incident, how do you feel? Let's explain it in pictures. Then he would ask us why it was like this. Choose one musical instrument to sit in a group, depending on what you're getting. Then he would have you tap in rhythm. (Cl.MS.1)

About a week after the incident happened, MC at Teams went to ask for information and provide an activity, to sit in a group and write down our feelings. Whatever we wanted. (Cl.MS.6)

##### 5.3.3.2 Medical Support

Prescribing medications alleviated symptoms like insomnia. Participants mentioned:

I went to the hospital to consult about what to do. The doctor recommended some medicine to help us sleep. Just take seven small pills. Otherwise, I don't take them. I cannot remember the doctor's name. It has been many months since the incident. (Cl.MS.1)

##### 5.3.3.3 Emotional Encouragement

Support from healthcare teams, families and religious networks was instrumental in recovery. Participants expressed:

Encouragement from colleagues and also groups from the network of monks that we work with. Most have support from their families. (Cl.MS.7)

##### 5.3.3.4 Resource Allocation and Prioritisation

Prioritising Care for the Severely Injured: Recognising the burden on healthcare providers and prioritising assistance for those most in need. Participants observed:

That day, I thought that the medical personnel of Maharaj Hospital in this province were already burdened. Military and police units that came to help were shot and injured as well as others. They provided full service there. I do not want help for myself. Let them help those who are hurt more than us. (Cl.MS.2)

These sub‐themes help to categorise the various forms of assistance provided by healthcare teams to victims of mass shootings, offering a structured approach to understanding the interventions and support mechanisms in place.

## 6 Discussion

This study interpreted the lived experiences of Thai individuals affected by public mass shootings during the COVID‐19 pandemic, shedding light on how individuals navigated existential fear, relational disruption and embodied trauma amid a context of heightened societal instability. Thematic analysis generated five core interpretive themes: *dwelling in the shadow of threat, bearing witness to collective rupture, embodied echoes of trauma, grounding the self through everyday rituals, and yearning for attuned care*.

*Biological consequences* surfaced as somatic symptoms—such as sleep disturbances, fatigue and heightened sensory awareness—reflecting the body's physiologic response to traumatic stress. These findings align with Roy's Regulator subsystem (C. Roy 2009) and are consistent with prior studies showing that trauma survivors often experience chronic sleep disruption and hyperarousal (Glasofer and Laskowski‐Jones 2018; Lowe and Galea 2017).

*Psychological responses* include hypervigilance, avoidance and emotional withdrawal, consistent with the Cognator subsystem in Roy's Adaptation Model. These findings echo previous research on mass shooting survivors, which reported elevated rates of PTSD and anxiety (Bharadwaj et al. 2021; DeLong 2021). However, this study adds nuance by highlighting culturally embedded coping strategies such as mindfulness and spiritual practices, which are less emphasised in Western literature (Khatib et al. 2022).

*Social consequences* were marked by disrupted roles, financial strain and an intensified sense of collective vulnerability. These findings support the interdependence mode of RAM and are consistent with studies showing that trauma can fracture social networks while simultaneously fostering solidarity (Newnham et al. 2020; Shultz et al. 2014). The theme bearing witness to collective rupture uniquely illustrates how shared trauma can generate communal resilience, a finding that contrasts with literature emphasising individual isolation post‐trauma (Lowe and Galea 2015).

*Participants employed diverse coping strategies*—ranging from recreational routines and familial bonding to expressive therapies and pharmacologic support. These responses reflect adaptive patterns across all four RAM modes and align with trauma‐informed care frameworks. The use of art therapy and journaling illustrates the therapeutic value of creative expression in trauma recovery (Efodzi et al. 2024).

Notably, this study highlights the variability of adaptive responses varied across individuals, from emotional withdrawal to ritualised engagement, Reinforcing Roy's conceptualization of adaptation as contextually mediated and dynamic. By

**framing** these narratives within the Roy Adaptation Model, the study contributes a culturally grounded perspective to the global discourse on trauma recovery and underscore the imperative for relationally attuned, trauma‐sensitive nursing interventions that support survivors' biopsychosocial needs and foster resilience across multiple domains of human adaptation.

## 7 Conclusions

This study underscores the profound psychological, social and embodied toll of mass shootings experienced during a global public health crisis. Survivors' narratives revealed how meaning was constructed through fear, connection and adaptive practices. Integrating these insights into trauma‐informed nursing care can enhance survivor recovery by validating emotional experience, supporting coping and reinforcing relational safety. These findings call for comprehensive, context‐responsive interventions across nursing, health policy and community‐based care systems.

### 7.1 Implications for Nursing and Health

The findings of this study present critical implications across health policy, clinical practice and future nursing research—particularly within trauma‐informed mental health care frameworks.

#### 7.1.1 Education Implications

This study highlights the urgent need to revise the nursing curriculum to include trauma‐informed care competencies specific to mass trauma events. Educational programmes should incorporate modules on psychological triage, survivor‐centred communication and culturally responsive interventions. Simulation‐based training and reflective practice should be used to prepare nursing students to respond effectively to trauma survivors, particularly in high‐impact events such as mass shootings.

#### 7.1.2 Policy Recommendations

Effective response to mass trauma must include intersectoral public health strategies. Policymakers should prioritise trauma‐sensitive preparedness training for first responders, law enforcement and healthcare workers, including mental health triage, safe evacuation protocols and psychological first aid. Public education campaigns such as ‘Run, Hide, Fight’ initiatives can empower community‐level resilience. Moreover, long‐term governmental support—financial, psychosocial and rehabilitative—is vital for addressing survivors' multidimensional needs and mitigating chronic morbidity.

#### 7.1.3 Practice Implications

Nurses play a central role in addressing the immediate and long‐term effects of trauma. Trauma‐informed care should validate fear, promote adaptive coping, and establish a foundation of relational and environmental safety. Interventions may include expressive therapies, stress‐reduction techniques and psychoeducation embedded in primary and community health services. Outcomes to monitor may include:

○ Phenomenological insights can further inform trauma‐informed nursing care by emphasising the existential dimensions of suffering and the need for holistic interventions (Al Kalaldeh et al. 2018).
○ Reduced PTSD symptomatology (e.g., via the PTSD Checklist for DSM‐5).
○ Improved sleep quality (reported or digitally tracked)
○ Re‐engagement in community and family roles

#### 7.1.4 Research Directions

This study reveals that while some psychological responses abate over time, unresolved fear and disrupted meaning systems may persist. Further research is needed to:

○ Explore longitudinal trajectories of psychological adaptation post‐mass trauma.
○ Design and evaluate rapid‐response nursing interventions tailored for civilian trauma.
○ Investigate the role of culturally rooted practices—spirituality, familial rituals and community gatherings—in enhancing resilience.

Embedding these insights into nursing education, practice and policy can contribute to more responsive, context‐sensitive systems of care for trauma‐exposed populations.

## Author Contributions

Ek‐Uma Imkome was solely responsible for the conception and design of the study, including the development of research questions and methodology and conducted all data collection, including participant recruitment, interviews and data management. The author performed all data analysis and interpretation and drafted the manuscript, including the literature review, results and discussion sections. The author ensured adherence to ethical guidelines and managed all administrative tasks related to the study. The author reviewed and approved the final version of the manuscript for publication.

## Funding

This work was supported by the Faculty of Nursing, Thammasat University (2563).

## Consent

The author obtained written and verbal informed consent from all participants to use their data in this study.

## Conflicts of Interest

The author declares no conflicts of interest.

## Data Availability

The data supporting this study are available on Figshare: https://doi.org/10.6084/m9.figshare.25018022.v2. These data are accessible under the terms of the Creative Commons Attribution 4.0 International licence (CC‐BY 4.0).
